# Syntheses of cyclic polylactides and the problem of catenane formation†

**DOI:** 10.1039/d4ra08683j

**Published:** 2025-02-05

**Authors:** Hans R. Kricheldorf, Steffen M. Weidner

**Affiliations:** a Universität Hambung, Institut für Technische und Makromolekulare Chemie Bundesstraße 45 D-20146 Hamburg Germany hrkricheldorf@aol.de; b Bundesanstalt für Materialforschung- BAM Richard-Willstätter-Straße 11 12489 Berlin Germany

## Abstract

Cyclic poly(l-lactide)s (PLAs) were prepared in bulk either by ring-expansion polymerization (REP) or by ring-opening polymerization (ROP) with simultaneous polycondensation (ROPPOC). In contrast to REP the latter method involves formation of linear chains and thus, may involve formation of polydisperse catenanes that affect crystallization. The reprecipitated PLAs were annealed at 120 °C and compared with regard to melting temperature (*T*_m_) and melting enthalpy (Δ*H*_m_). For similar molar masses the PLAs prepared by REP and ROPPOC had almost identical *T*_m_'s and crystallinities. Furthermore, the influence of REP and ROPPOC catalysts on the morphology of the virgin reaction products was compared.

## Abstract 2

Cyclic poly(l-lactide)s (PLAs) were prepared in bulk either by ring-expansion polymerization (REP) or by ring-opening polymerization (ROP) with simultaneous polycondensation (ROPPOC). No evidence of substantial catenane formation was found.

## Graphical abstract

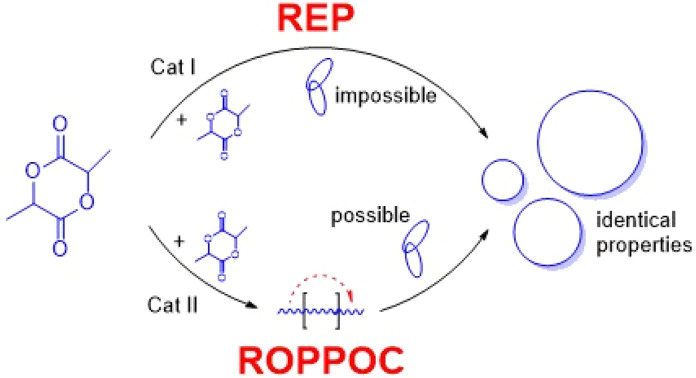

## Introduction

In his first theory of irreversible step-growth polymerization (SGP) in 1946, Flory postulated, based on calculations of chain conformations, that the chain ends of polymers never meet, so that end-to-end cyclization (ete) does not occur.^1^ A few years later, Jacobson and Stockmayer published a first theory of reversible SGPs in which they accepted Flory's dogma also for reversible polycondensations (revPOCs), but postulated the formation of cyclic oligomers and low molar mass polymers *via* “back-biting” of the most active chain end.^2,3^ They pointed out that the existence of ring-chain equilibration automatically implies ring–ring and chain–chain equilibration, so that any revPOC represents a thermodynamically controlled process at any stage of the polymerization, provided that the equilibration is fast enough relative to the progress of the polycondensation.

**Fig. 1:**
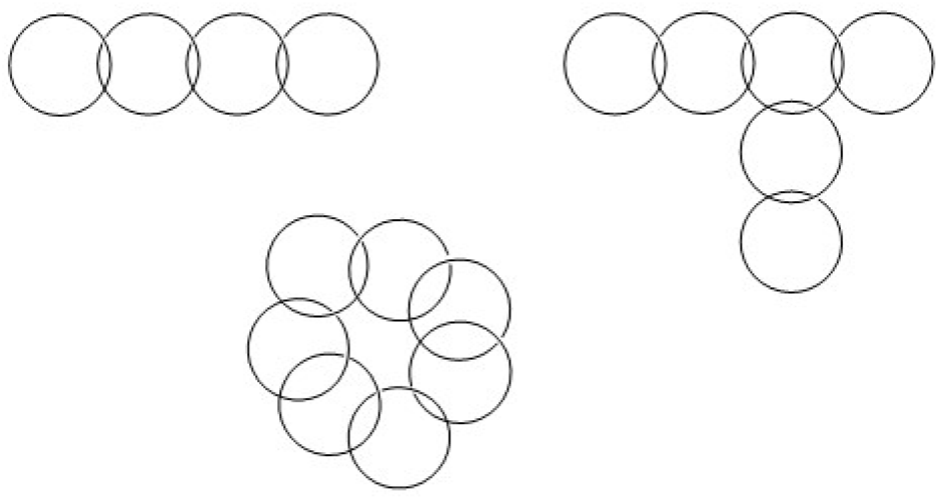
Schematic examples of disperse catenanes.

After 1970, Flory's dogma was disproved, first by new mathematical treatments of irreversible polycondensations by Gordon *et al.*^4,5^ and by Stepto *et al.*^6,7^ After the matrix-assisted laser desorption/ionization (MALDI) time of flight (TOF) mass spectrometry became commercially available, the experimental evidence was contributed by the first author and coworkers.^8,9^ More recently, the authors demonstrated that ete-cyclization also plays a major role in revPOCs.^10,11^ The fraction of cycles increases with higher conversions and can reach 100% at 100% conversion. The existence of ete-cyclization in both irrevPOCs and revPOCs raises the interesting question of the extent to which bulk step-growth polymerizations (SGP) involve the formation of catenanes resulting from the threading of linear chains through preformed cyclic polymers. Catenanes formed under such conditions do not necessarily consist of a quasi-linear arrangement of several cycles but can also adopt 2D and 3D architectures (Fig. 1). All these different architectures have in common that they are much less favorable for crystallization than simple cycles.

**Scheme 1:**
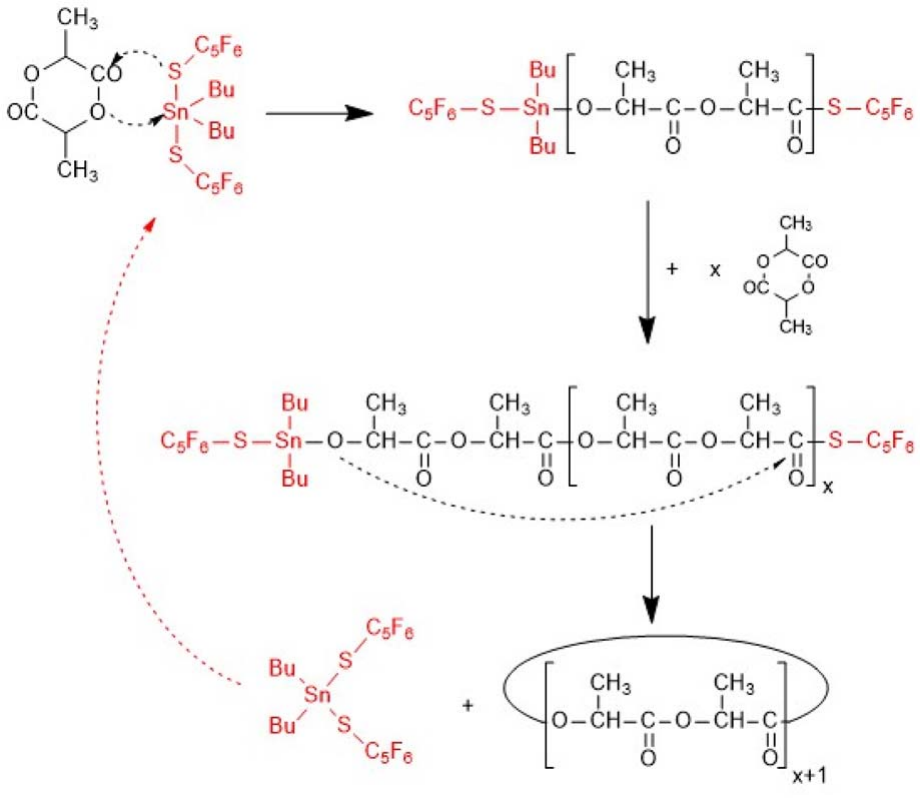
ROPPOC mechanism catalyzed by BuSnSPF.

Polycondensations with conversions >99.9% and almost quantitative cyclization can be achieved by ring-opening polymerization (ROP) combined with simultaneous polycondensation (including ete-cyclization), a so-called ROPPOC process (see Scheme 1 and ref. 12).

**Scheme 2:**
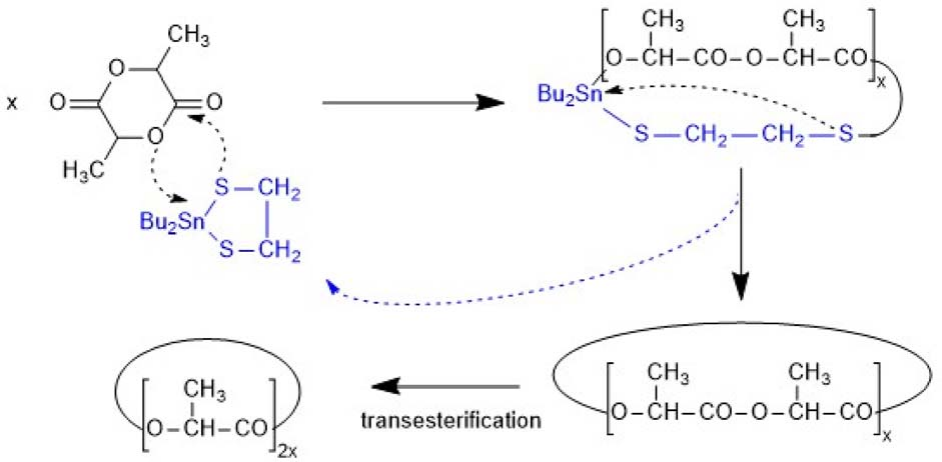
REP mechanism catalyzed by DSTL.

Cyclic polyesters with the same structure and ring size can also be prepared by ring-expansion polymerization using cyclic tin catalysts (Scheme 2).^13–16^

However, in contrast to ROPPOC, catenanes cannot be formed because linear chains are never involved in REP using cyclic covalent catalysts. Therefore, a comparison of cyclic polyesters prepared *via* ROPPOC and REP under similar conditions could provide indirect evidence for the formation of disperse catenanes in the ROPPOC process, as they reduce the melting temperature (*T*_m_) and melting enthalpy (Δ*H*_m_) of the whole polyester. Therefore, one aim of this work was to prepare cyclic poly(l-lactide)s produced in bulk *via* ROPPOC and REP under identical conditions and with similar molar masses. An overriding trend towards lower *T*_m_'s and Δ*H*_m_'s for all ROPPOC products can then be interpreted as indirect evidence of “contamination” with catenanes.

A second purpose of this work was to compare REP and ROPPOC catalysts with respect to the effects of annealing in the presence of the catalysts. It has recently been shown that annealing of cyclic PLAs at 140 °C in the presence of different REP catalysts can favor the formation of extended-ring crystallites in the mass range of *m*/*z* 3000–15 000.^17,18^ Smoothing their surface by transesterification produces a saw-tooth pattern in the MALDI-TOF mass spectra, indicating a narrow distribution of ring sizes in the individual crystallites. The whole process is slow and represents a thermodynamic optimization of the crystallites resulting in high *T*_m_'s (typically >195 °C) and high Δ*H*_m_'s (typically >95 J g^−1^). The question to be answered is whether ROPPOC catalysts of similar reactivity will induce an analogous modification of PLA crystallites.

Finally, it should be mentioned that numerous research groups have attempted to synthesize individual catenanes by directed stepwise synthesis. Such strategies are beyond the scope of this work, but review articles covering this area of work are mentioned.^19–21^

## Experimental

### Materials

l-Lactide (Corbion-Purac, Netherlands) was kindly supplied by Thyssen-Uhde (Berlin, Germany) and recrystallized from toluene (99.98%, extra dry, Thermo-Fisher Scientific, Schwerte, Germany). Anhydrous dichloromethane was purchased from Thermo-Fisher (Schwerte, Germany).

**Scheme 3:**
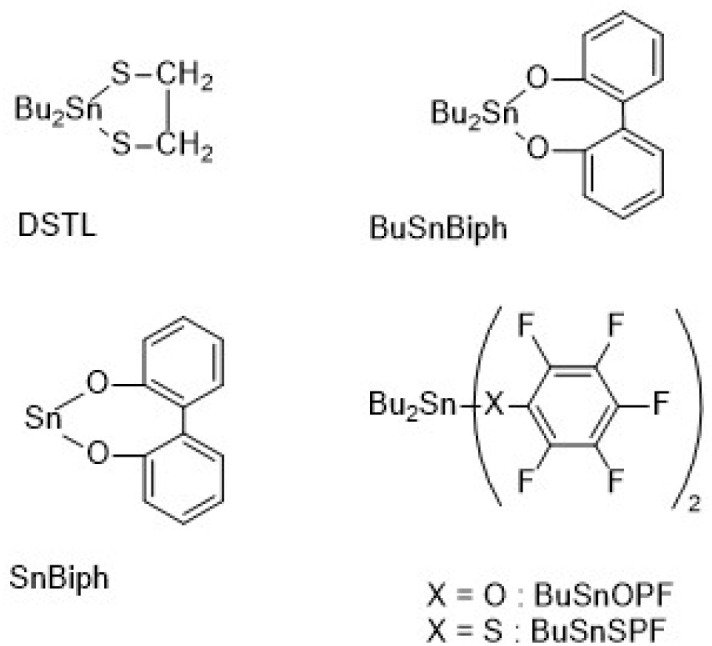
Formulas and acronyms of catalysts used in this work.

The catalysts defined in Scheme 3 were synthesized as previously described: DSTL (2,2-dibutyl-2-stana-1,3-ditholane)^13^ SnBiph (2-stanna-1,3-dioxa-4,5,6,7-dibenzepane),^22^ BuSnBiph (2,2-dibuty-2-stanna-1,3-dioxa-4,5,6,7-dibenzepane),^23^ BuSnSPF (dibutyltin bis(pentafluorothiophenolate)),^15^ BuSnOPF (dibutyltin bis(pentafluorophenoxide)).^24^

### Polymerizations catalyzed by DSTL or BuSnSPF

Catalyst (0.2 or 0.08 mmol) and l-lactide (40 mmol) were weighed into a flame-dried 50 mL Erlenmeyer flask under an argon blanket. The reaction vessel was immersed in an oil bath heated at 140 °C. After 2 d, the reaction vessel was destroyed, and the crystalline plaque of PLA was broken into pieces. One small piece was used for characterization and another piece was used for additional annealing (under argon) at 140 °C for 4 d. The remaining PLA was dissolved in dry dichloromethane (approximately 70 mL) and precipitated in ligroin (700 mL). After drying, the PLA was dissolved again in dichloromethane (*ca.* 25 mL) in a 50 mL round bottom flask and gradually heated to 120 °C. After evaporation of the dichloromethane (*ca.* 30–40 min), the PLA began to crystallize. Annealing was continued for 24 h. The resulting crystalline PLA was broken into four pieces. Two were used for characterization and the other two were annealed at 120 °C for another day.

### Polymerizations catalyzed by SnOct_2_, SnBiph, BuSnOPF or BuSnBiph

The catalyst (0.08 mmol) and l-lactide (40 mmol) were weighed into a flame-dried 50 mL Erlenmeyer flask under an argon blanket and a magnetic bar was added. The reaction vessel was placed in an oil bath thermostated at 160 °C. The reaction mixtures became viscous within 1 h, and after 3 h the reaction products were cooled to 25–30 °C. Dichloromethane (approximately 50 mL) was added to dissolve the PLAs. The resulting solutions were diluted with 10 mL of dichloromethane and precipitated in ligroin (about 600 mL). The precipitated PLA was dried at 60 °C *in vacuo*. Approximately 4 g of the cyclic PLAs were then dissolved in dichloromethane (25 mL) and slowly immersed in an oil bath heated to 120 °C. After evaporation of the dichloromethane (30–40 min), the remaining PLA began to crystallize. Annealing at 120 °C was continued for 23 h. Afterwards, the solid foams were isolated by destroying of the reaction vessels and cut into four pieces. Two pieces were annealed at 120 °C for another day and the other pieces were used for characterization.

### Measurements

The MALDI TOF mass spectra were recorded with an Autoflex maX mass spectrometer (Bruker Daltonik GmbH, Bremen, Germany). All spectra were measured in the linear positive ion mode. The MALDI targets were prepared from chloroform solutions of poly(l-lactide) (3–5 mg mL^−1^). Potassium trifluoroacetate (2 mg mL^−1^ in tetrahydrofuran) was used to enable potassium adduct ion formation. 20 μL of the sample solution, 2 μL of the salt solution and 50 μL of a matrix solution of *trans*-2-[3-(4-*tert*-butylphenyl)-2-methyl-2-propenylidene] malononitrile (DCTB, 20 mg mL^−1^ in CHCl_3_) were pre-mixed in an Eppendorf vial. Finally, 1 μL of the combined solution was dropped onto the MALDI target. Data were recorded and evaluated using the manufactures software (FlexControl/FlexAnalysis).

The size exclusion chromatography (SEC) measurements were performed in a modular system kept at 40 °C consisting of an isocratic pump, 1 mL min^−1^ and a refractive index detector (RI-501-Shodex). Samples were injected manually (100 μL, 2–4 mg mL^−1^). For instrument control and data calculation WinGPC software (PSS, Mainz – now part of Agilent Technologies) was used. The calibration was performed using polystyrene standard sets (Agilent Technologies, Mainz).

The differential scanning calorimetry (DSC) heating curves were recorded on a DSC-1 (Mettler-Toledo, Germany) after fresh calibration with indium and zinc at a heating rate of 10 K min^−1^. Only the first heating curves were evaluated. Instrument control and data calculation were performed using the instrument Star Software-11.

## Results and discussion

### Polymerizations catalyzed by BuSnSPF (Table 1) and DSTL (Tables 2 and 3)

Initially, two sets of experiments were carried out using BuSnSPF as the ROPPOC catalyst and DSTL as the REP catalyst, as the Sn–S groups are less basic and therefore less prone to side reactions than the Sn–O group. The starting materials were prepared by bulk polymerization at 140 °C, as it was recently found that DSTL gives higher molar masses at 140 °C than at 160 °C.^17^ The PLAs isolated after 2 d were characterized in terms of molar masses, *T*_m_ and Δ*H*_m_ (Tables 1 and 2).

**Table 1:** Cyclic PLAs prepared with BuSnSPF in bulk at 140 °C

Exp. no.	LA/Cat	*T* (°C)	Time (d)	*M*_n_ (g mol^−1^)	*M*_w_	*T*_m_ (°C)	Δ*H*_m_ (J g^−1^)
1A^a^	200/1	140	2	85 000	251 000	194.3	97.6
1B	200/1	140	6	37 000	93 000	190.4	100.3
2A^b^	200/1	120	1	106 000	256 000	180.6	57.6
2B^b^	200/1	120	2	57 000	123 000	181.6	75.3
3A^a^	500/1	140	2	132 000	305 000	195.8	90.6
3B	500/1	140	6	57 000	131 000	194.3	73.3
4A^b^	500/1	120	1	138 000	321 000	182.3	54.5
4B^b^	500/1	120	2	48 000	114 000	181.4	73.8

a Synthesis of the starting material.

b Annealing for 1 d at 120 °C after precipitation of the starting material.

**Table 2:** Cyclic PLAs prepared with DSTL in bulk at 140 °C

Exp. no.	LA/Cat	*T* (°C)	Time (d)	*M*_n_ (g mol^−1^)	*M*_w_	*T*_m_ (°C)	Δ*H*_m_ (J g^−1^)
1A^a^	200/1	140	2	128 000	345 000	194.4	92.7
1B^b^	200/1	140	6	45 000	110 000	196.0	98.6
2A^b^	200/1	120	1	116 000	320 000	180.5	45.8
2B^b^	200/1	120	2	73 500	201 000	180.8	69.0
3A^a^	500/1	140	2	122 000	350 000	196.1	95.6
3B^b^	500/1	140	6	—	—	192.5	100.1
4A^b^	500/1	120	1	109 000	302 000	181.4	56.6
4B^b^	500/1	120	2	61 000	160 000	180.9	71.0

a Synthesis of the starting material.

b Annealing for 1 d at 120 °C after precipitation of the starting material.

The SEC data presented in Tables 1 and 2 (no. 1A and B) showed that both catalysts yielded high molar masses with weight average molar masses (*M*_w_'s) above 250 000 g mol^−1^. In addition, a significant decrease in molar masses was observed after annealing for 6 d in both cases. Therefore, the PLAs prepared by either BuSnSPF or DSTL were comparable.

The DSC measurements of all samples were carried out at least twice or even three times from different locations of the same crystalline plaque, as it was found in previous studies that larger samples of crystalline PLA were not perfectly homogeneous in terms of perfection of crystallites and spherulites, even when annealed for 1 d or more. All DSC measurements are presented in tables in the ESI (Tables S1 and S2†). However, for ease of comparison and discussion, only the highest *T*_m_ and Δ*H*_m_ values are shown in the tables presented in the text. These data clearly show that there is no significant difference between the PLAs prepared with the two catalysts. In fact, the crystallinities of the samples prepared with the ROPPOC catalyst were slightly higher than those of the DSTL-catalyzed samples, clearly indicating that the PLAs prepared with BuSnSPF did not contain a higher fraction of amorphous material.

**Fig. 2:**
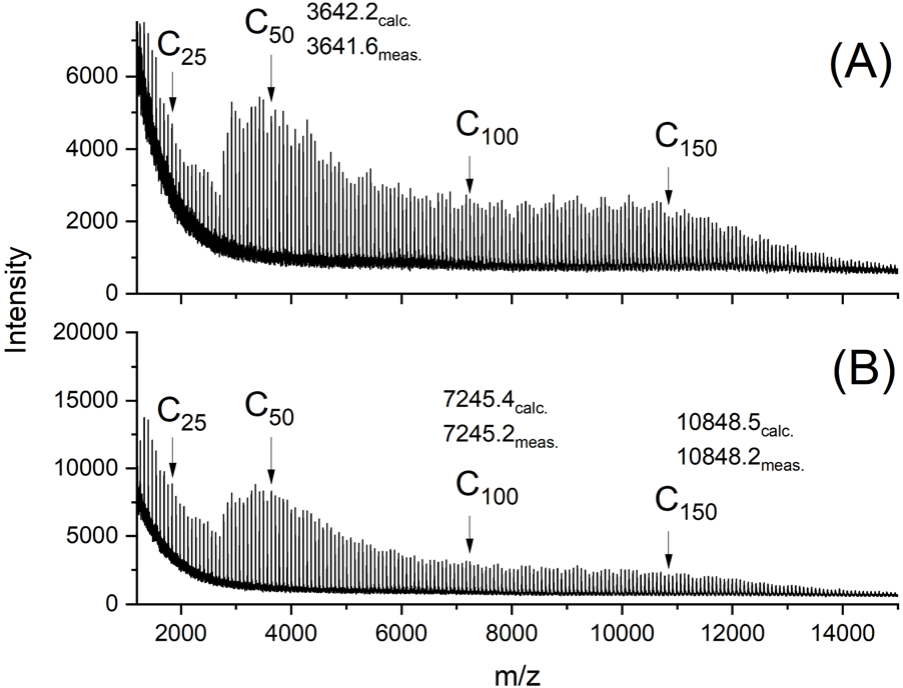
MALDI TOF mass spectra of cyclic PLAs prepared with BuSnSPF at 140 °C/2 d: (A) LA/Cat = 200/1 (no. 1A, Table 1), (B) LA/Cat = 500/1 (no. 3A, Table 1).

**Fig. 3:**
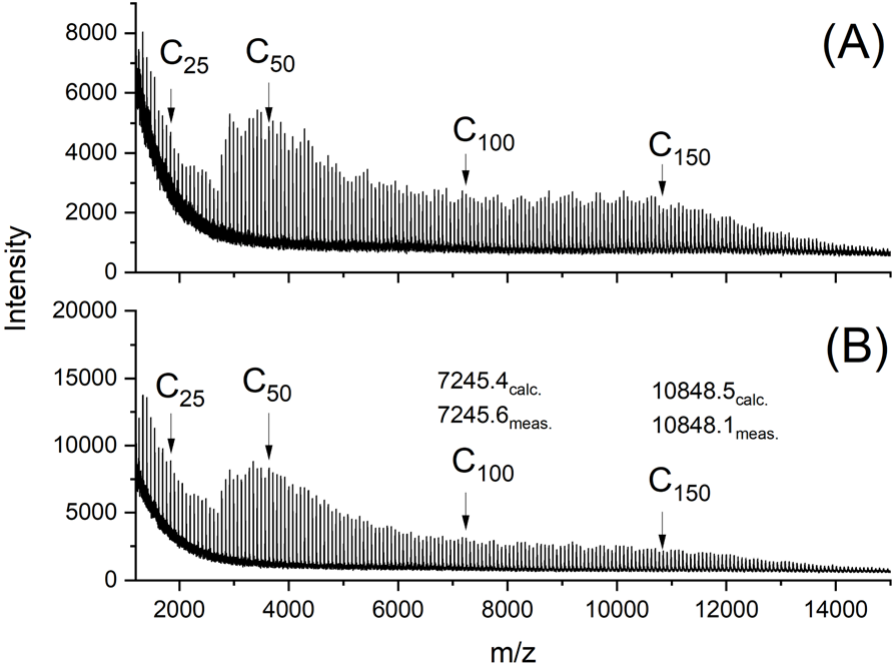
MALDI TOF mass spectra of cyclic PLAs prepared with DSTL at 140 °C/2 d (no. 1A, Table 2), (B) LA/Cat = 500/1 (no. 3A, Table 2).

**Fig. 4:**
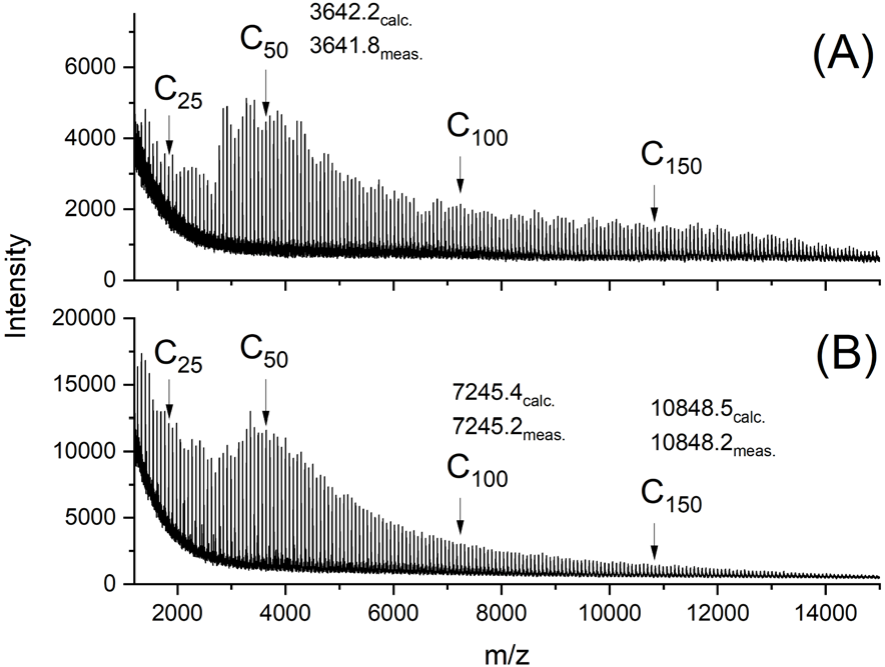
MALDI TOF mass spectra of cyclic PLAs prepared with BuSnSPF after annealing at 120 °C/1 d: (A) LA/Cat = 200/1 (no. 2A, Table 1), (B) LA/Cat = 500/1 (no. 4A, Table 1).

**Fig. 5:**
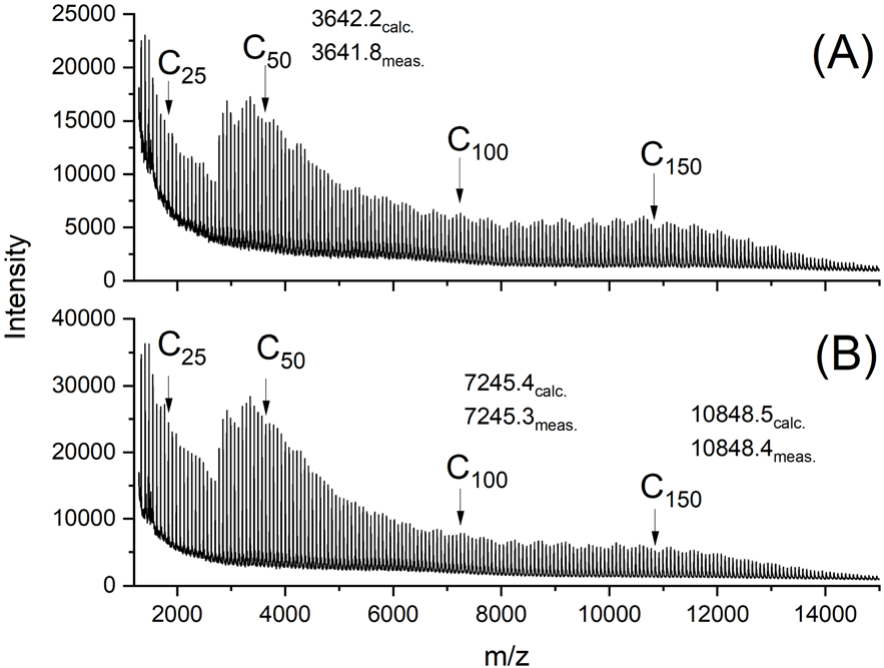
MALDI TOF mass spectra of cyclic PLAs prepared with DSTL after annealing at 120 °C/1 d: (A) LA/Cat = 200/1 (no. 2A, Table 2), (B) LA/Cat = 500/1 (no. 4A, Table 2).

**Fig. 6:**
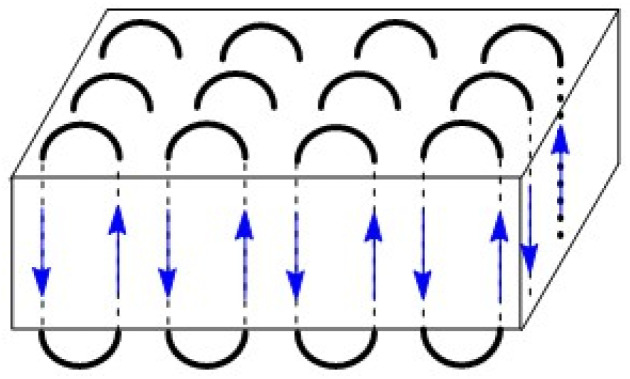
Two-dimensional scheme of an extended-ring crystallite.

The MALDI-TOF mass spectra were also nearly identical and showed mass peaks of cycles detectable up to *m*/*z* 15 000 to 16 000 (Fig. 2–5). In addition, the so-called “sawtooth pattern” (STP) first reported and explained in two recent publications was clearly observed in the spectra of all four samples.^25,26^ This sawtooth pattern is characteristic of cyclic PLAs in the mass range of *m*/*z* 3000–15 000. In this mass range, cyclic PLA crystallites are built up by extended rings (Fig. 6). This (previously published) interpretation^25,26^ is based on the following observations. First, STP has never been observed for linear PLAs, regardless of reaction conditions. Second, STP has never been observed for cyclic PLAs quenched from the melt. Third, STP only forms as a consequence of annealing in the presence of an active transesterification catalyst (the commercial PLAs contain a catalyst poison). Fourth, the lengths of the extended rings are consistent with the thickness of the crystallites as determined by SAXS. Fifth, an STP appears in combination with a new maximum in the molecular weight distribution, indicating that the cycles formed in the solid state are the result of a thermodynamic optimization process. Sixth, extended-ring crystallites represent a thermodynamic optimum for the mass range below 2000 Da for the following reasons. (A) All linear segments necessarily adopt an antiparallel orientation typical of the α-modification of PLA. (B) There are no defects due to burial of chain ends within the crystal lattice. (C) A “tooth” represents crystallites composed of rings with nearly identical ring size. Thus, the loops on either side of the crystallite are nearly equal in size, resulting in a thermodynamically optimized smooth surface. The sawtooth pattern results from thermodynamically controlled surface modification of the extended ring crystallites *via* transesterification reactions in the presence of a reactive transesterification catalyst. The mass spectra of Fig. 2A and 4A are the first examples of cyclic PLAs with a sawtooth pattern prepared in bulk with a ROPPOC catalyst. Two trends are noteworthy. First, the saw-tooth pattern was slightly more pronounced when DSTL was used as the catalyst. Regardless of the catalyst, it was more pronounced when the catalyst concentration was higher ((A) spectra), consistent with the hypothesis that this pattern is a consequence of catalyzed transesterification reactions over the surface of the crystallites.

Eventually, the PLA samples isolated after 2 d were dissolved in dry dichloromethane and precipitated in ligroin to remove most of the catalyst. The resulting PLAs were again dissolved in dichloromethane and heated to 120 °C to evaporate the solvent and induce crystallization. A fraction of the solid PLAs was then isolated after 1 d and a second fraction after 2 d. The temperature of 120 °C was chosen because it was known from DSC heating traces of amorphous PLA that crystallization becomes rapid around 110 °C, and above 120 °C PLA crystallizes in the most thermodynamically stable α-modification regardless of whether the molar masses are low or high and regardless of whether the topology is linear or cyclic.^27,28^ By annealing the PLAs at 120 °C for 1 d and 2 d, kinetic effects and differences in crystal modification should be excluded.

The mass spectra obtained after annealing at 120 °C for 1 d were nearly identical to those of the starting materials and showed the sawtooth pattern (Fig. 4 and 5). It was known from previous studies that 120 °C and reaction times less than 2 d were not sufficient to produce a sawtooth pattern. Therefore, the mass distributions shown in Fig. 4 and 5 simply indicated that the mass distributions of the starting materials were not significantly altered by precipitation and annealing at 120 °C.

An additional series of DSTL-catalyzed polymerizations was performed at 140 °C in concentrated solutions of aromatic solvents (Table 3), because these conditions were found in a previous work to give rather high molar mass cyclic PLAs with high catalyst concentration. The samples prepared at 140 °C were precipitated into ligroin and annealed at 120 °C quite analogous to the conditions used for the samples listed in Table 2. Despite the different conditions used for the syntheses the DSC data show a satisfactory agreement with those listed in Table 2.

**Table 3:** Cyclic PLAs prepared with DSTL (LA/Cat = 50/1) in 2 M solution

Exp no.	Solvent	*T* (°C)	*T* (d)	*M*_n_ (g mol^−1^)	*M*_w_	*T*_m_ (°C)	Δ*H*_m_ (J g^−1^)
1^a^	*o*-Dichlorobenzene	140	1	85 000	215 000	—	—
2A^b^	*o*-Dichlorobenzene	120	1	40 000	124 000	174.7/176.0	56.3/53.7
2B^b^	*o*-Dichlorobenzene	120	2	34 000	105 000	174.2/175.5	65.7/63.5
3^a^	Anisole	140	4	—	—	—	—
4A^b^	Anisole	120	1	30 000	78 000	173.7/174.3	59.0/55.5
4B^b^	Anisole	120	2	25 500	59 000	172.7/173.6	66.2/62.6

a Synthesis of the starting material.

b Annealing at 120 °C after precipitation of the starting material.

Finally, a series of annealing experiments with PLA samples prepared with DSTL in a different context should be mentioned (partially reproduced in Table S3†).^17,18^ These samples were prepared with an LA/Cat ratio of 200/1, analogous to the experiments listed in Tables 1 and 2, and annealed at 140 °C. The Δ*H*_m_ values obtained for annealing times <7 d show a satisfactory agreement with the data presented in Table 2, thus, demonstrating a good reproducibility. Annealing for 7 d resulted in slightly higher Δ*H*_m_ values (100–102 J g^−1^) because, as discussed above, the catalyst left in these samples causes a smoothing of the crystallite surfaces by transesterification.

### Polymerizations catalyzed by SnOct_2_, SnBiph, BuSnOPF and BuSnBiph (Table 4)

In order to clarify whether or not the results obtained with the aforementioned Sn–S type catalysts can be generalized, experiments were carried out with two pairs of Sn–O type catalysts. The ROPPOC catalyst SnOct_2_ was compared with SnBiph as an example of tin(ii)-based catalysts, and the ROPPOC catalyst BuSnOPF was compared with the REP catalyst BuSnBiph. Since all four catalysts are quite reactive, polymerizations for 2 h at 160 °C were sufficient to achieve complete conversion (*i.e.* 97% for thermodynamic reasons).

The virgin PLAs were precipitated to remove most of the catalyst, and then the mainly amorphous PLAs were annealed at 120 °C for 1 or 2 d, analogous to the experiments performed with the Sn–S type catalysts described above. Each sample was subjected to two or three DSC measurements, which are summarized in Table S4 of the ESI.† These data illustrate the scatter of *T*_m_ and Δ*H*_m_ values resulting from measurements of two or three different locations of larger crystallized plaques (diameter about 2 cm). For simplicity, only the highest values are shown in Table 3.

High molar masses with *M*_w_ values above 280 000 g mol^−1^ were obtained for all starting materials, justifying a comparison of all samples. The most important result is the finding that after annealing for 2 d, which reduces the influence of kinetic effects, the crystallinity of the PLAs prepared by ROPPOC catalysts is slightly higher than that of the comparable REP catalysts (no. 2B *vs.* 4B or 6B *vs.* 8B, Table 4). This finding is in agreement with the results obtained by BuSnSPF and DSTL. In terms of *T*_m_, a slightly higher value was found for SnOct_2_ compared to SnBiph, but a slightly higher value for the BuSnBiph (REP) catalyst. However, these differences are not greater than 1.5 °C and fall within the range of scattering when different locations of the crystalline plaques are measured. Therefore, these *T*_m_ and Δ*H*_m_ measurements again do not indicate that the ROPPOC catalysts yield amorphous catenanes in amounts significantly above 5%.

**Table 4:** Cyclic PLAs prepared with SnOct_2_, SnBiph, BuSnOPF and Bu_2_SnBiph (LA/Cat = 500/1) in bulk at 160 °C

Exp. no.	Cat	*T* (°C)	Time (h)	Yield (%)	*M*_n_ (g mol^−1^)	*M*_w_	*T*_m_ (°C)	Δ*H*_m_ (J g^−1^)
1	SnOct_2_	160	3	93	81 000	287 000	—	—
2A		120	24	—	73 000	254 000	177.7	52.6
2B		120	48	—	66 000	195 000	179.5	65.8
3	SnBiph	160	3	94	212 000	505 000	—	—
4A		120	24	—	170 000	430 000	180.8	55.0
4B		120	48	—	126 000	335 000	178.3	64.7
5	BuSnOPF	160	3	93	134 000	325 000	—	—
6A		120	24	—	91 000	210 000	180.8	60.0
6B		120	48	—	55 000	142 000	180.0	68.0
7	BuSnBiph	160	3	93	132 000	283 000	—	—
8A		120	24	—	109 000	225 000	178.1	59.0
8B		120	48	—	65 000	165 000	181.7	62.9

### Discussion of catenane formation

**Fig. 7:**
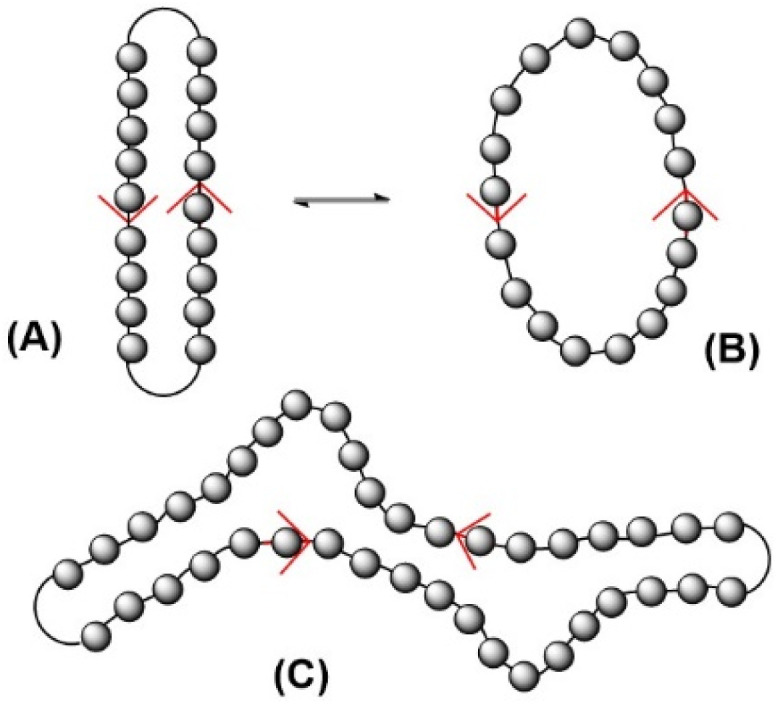
Equilibrium between perfect collapsed (A) and open conformation (B) of small cyclic PLAs and collapsed conformation of larger cycles with defects (C).

Finally, the question of whether or not the formation of catenanes is highly probable deserves discussion. In this context, the formation of extended ring crystallites is worth considering. Several research groups (including the authors) have reported that nucleation and crystal growth of cyclic polar polymers is faster than that of linear chains with similar molecular weights.^29–32^ This finding may be explained by the fact that the number of conformational changes required to adopt the collapsed conformation necessary for incorporation into the crystal lattice is much lower than in the case of linear chains of similar molecular weight. Furthermore, a relatively high fraction of cycles in collapsed conformation (A and C in Fig. 7) should be present in the melt or concentrated solutions for the following reasons. The widening of the cycle (A → B in Fig. 7) is accompanied by a gain in entropy, but unlike the melting process, there is no gain in translational motion. The limited gain in rotational motion is accompanied by only a small gain in entropy. In contrast, the parallelization of chain segments involves a rather large enthalpy gain due to the dense packing of antiparallel oriented dipole moments. The 3D structure of the α-modification of PLA shows that this orientation is a thermodynamically favorable conformation. PLA like polyglycolide have the highest density of polar (OCO) groups along the chain and these polymers have the highest melting temperatures of all aliphatic polyesters (200–201 °C for annealed cyclic PLAs). These high melting endotherms represent a high ratio of melting enthalpy to entropy. If the expansion and collapse of cyclic PLAs is understood as a 2-D model of the melting/crystallization process, it is understandable why collapsed conformations largely predominate in molten PLA.

In the case of large cycles, the collapsed conformation is certainly not perfect for the entire cycle, and defects may be present, as shown in structure C of Fig. 7. However, even such a collapsed conformation with defects is a serious obstacle to threading through the cycles. Theoretical calculations and computer simulations concerning the conformations of cyclic polymers have been published by several authors^33,34^ and they suggest that cyclic polymers have a high tendency to adopt more or less collapsed or compact conformational states, which supports our hypothesis. This hypothesis combines information from the crystallization of cycles with information from the crystal lattice with the results obtained in this study and suggests the conclusion that the formation of catenanes is a rather rare event in the synthesis of polar cyclic polymers. However, this hypothesis certainly requires further experimental and theoretical studies for its confirmation.

## Conclusions

The results of this work allow the following conclusions: first, under identical conditions, REP and ROPPOC catalysts yield cyclic PLAs with almost identical thermal properties such as *T*_m_ and Δ*H*_m_. Second, regardless of whether Sn–S or Sn–O type catalysts were used, there is no evidence that amorphous disperse catenanes were formed in quantities greater than 5% in ROPPOC type polymerizations. Third, ROPPOC catalysts yield extended ring crystallites that show a “saw tooth” pattern in the mass spectra very similar to REP catalysts.

## Data availability

Data for this article are available at Zenodo (DOI: https://doi.org/10.5281/zenodo.14731603).

## Author contributions

HRK – conceptualization, investigation, methodology, project administration, writing – original draft, SMW – data curation, investigation, methodology, visualization, writing – review & editing.

## Conflicts of interest

There are no conflicts to declare.

## Supplementary Material

RA-015-D4RA08683J-s001
