# Supplementary material for: Syntheses of cyclic polylactides and the problem of catenane formation
Source: RSC Adv. 2025 Feb 5;15(5):3686–92. doi: 10.1039/d4ra08683j (PMC11795257; doi:10.1039/d4ra08683j)
Supplement: RA-015-D4RA08683J-s001 [file RA-015-D4RA08683J-s001.pdf]

This journal is © The Royal Society of Chemistry 2025

Supplementary Information

to

Syntheses of Cyclic Polylactides and the Problem of Catenane Formation

Hans R. Kricheldorf[a)] and Steffen M. Weidner[b)]

a) Institut für Technische und Makromolekulare Chemie, Bundesstr. 45, D-20146 Hamburg, Germany

b) Bundesanstalt für Materialforschung und -prüfung (BAM), Richard Willstätter Str. 11, D-12489 Berlin, Germany

Table S1 Cyclic PLAs prepared with BuSnSPF in bulk at 140 °C

Exp. No.	LA/Cat	Temp. (°C)	Time (d)	M_n (g mol^{-1})	M_w	T_m (°C)	ΔH_m (Jg^{-1})
1A[a)]	200/1	140	2	81 000	250 000	194.3	97.6
1B	200/1	140	6	37 000	93 000	190.4	100.3
2A[b)]	200/1	120	1	106 000	256 000	179.4/180.6	52.2/57.6
2B[b)]	200/1	120	2	57 000	123 000	181.6/181.7	75.3/74.7
3A[a)]	500/1	140	2	132 000	305 000	195.8	90.6
3B	500/1	140	6	57 000	131 000	194.3	73.3
4A[b)]	500/1	120	1	138 000	321 000	181.1/182.3	51.5/54.5
4B[b)]	500/1	120	2	48 000	114 000	180.3/181.4	73.2/73.8

a) Synthesis of the starting material, b) annealing at 120°C after precipitation of the starting material

Table S2 Cyclic PLAs prepared with DSTL at 140 °C

Exp. No.	LA/Cat	Temp. (°C)	Time (d)	M_n (g mol^{-1})	M_w	T_m (°C)	ΔH_m (Jg^{-1})
1A[a]	200/1	140	2	130 000	345 000	194.4	92.7
1B[a]	200/1	140	6	45 000	110 000	196.0/195.2	98.6/97.3
2A[b]	200/1	120	1	116 000	320 000	180.5/179.9	35.8/45.8
2B[b]	200/1	120	2	73 500	201 000	180.8/179.8	69.0/66.8
3A[a]	500/1	140	2	122 000	350 000	196.1	95.6
3B[a]	500/1	140	6	-	-	192.5/192.5	100.1/99.2
4A[b]	500/1	120	1	109 000	302 000	180.3/181.4	56.6/54.7
4B[b]	500/1	120	2	61 000	160 000	180.4/180.9	71.0/69.6

a) Synthesis of the starting material, b) annealing at 120°C after precipitation of the starting material

Table S3 REP conducted in bulk at 140 °C catalyzed by DSTL (LA/Cat =200/1)[a)]

Exp. No.	Time (d)	M_n (g mol^{-1})	M_w	T_m (°C)	ΔH_m (Jg^{-1})
1A	2	64 000	153 000	194.7	94.8
1B	3	121 000	305 000	197.2	99.9
1C	4	198 000	387 000	199.1	101.2
1D	7	98 000	243 000	199.7	102.6
2A	2	112 000	305 000	192.5	93.6
2B	2	91 000	264 000	199.1	101.2

a) Data reproduced from refs. 17 and 18

Table S4 Cyclic PLAs prepared with four different catalysts in bulk (LA/Cat = 500/1)

Exp. No.	Catalyst	Temp. (°C)	Time (h)	Yield (%)	M_n (g mol^{-1})	M_w	T_m (°C)	ΔH_m (Jg-1)
1[a]	$SnOct_2$	160	3	93	81 000	287 000	-	-
2A[b]		120	24	-	73 000	254 000	177.7/177.8	52.6/52.5
2B[b]		120	48	-	66 000	195 000	179.5/178.7	65.8/(62.3
3[a]	SnBiph	160	3	94	212 000	505 000	-	-
4A[b]		120	24	-	170 000	430 000	180.0/1808	54.1/55.0
4B[b]		120	48	-	126 000	335 000	177.7/178.3	64.7/64,1
5[a]	BuSnOPF	160	3	93	134 000	325 000	-	-
6A[b]		120	24	-	91 000	210 000	180.8/178.8	60.0/59.0
6B[b]		120	48	-	55 000	142 000	178.1/180.0	62.1/72.0
7[a]	BuSnBiph	160	3	93	132 000	283 000	-	-
8A[b]		120	24	-	109 000	225 000	177.7 178.1	56.0/59.0
8B[b]		120	48	-	65 000	165 000	180.5/181.7	57.1/62.9

a) Synthesis of the starting material, b) annealing at 120°C after precipitation of the starting material
